# Postural control and neuromuscular activation during balance in elite Chinese martial artists and sprinters

**DOI:** 10.3389/fphys.2025.1701020

**Published:** 2025-11-27

**Authors:** Huanhuan Zhu, Xiaobin Wei, Qingquan Song, Yifan Zhao, Hui Chen, Xiaoping Chen

**Affiliations:** 1 Sports Training Research Center, China Institute of Sport Science, Beijing, China; 2 Division of Sports Science and Physical Education, Tsinghua University, Beijing, China; 3 School of Physical Education, Qingdao University, Qingdao, China; 4 School of Sports and Health Management, Chongqing University of Education, Chongqing, China

**Keywords:** core stability, neuromuscular activation, FMS, vertical jump, Y balance tests

## Abstract

**Background:**

Postural control integrates core stability, coordination, and balance shaped by specific training. Sprinting emphasizes stretch–shortening-cycle power and trunk extensor use, whereas Chinese martial arts stress multi-planar core-hip control. Yet rigorous comparisons in elite athletes that pair functional performance with time-synchronized surface electromyography are scarce, limiting insight into how long-term training sculpts neuromuscular strategies when fundamental movement capacity appears similar. This study compares elite martial artists and sprinters to delineate differences in core stability, dynamic balance, explosive power, and task-specific muscle activation, informing conditioning and cross-training. Thirty-two first-level male athletes from Beijing Sport University (martial arts/sprinting, n = 16) participated in a cross-sectional study. Assessments included the Functional Movement Screen (FMS), Countermovement jump (CMJ), squat jump (SJ), and Y-Balance Test (YBT), administered in a Latin-square order. Surface Electromyography (sEMG) was collected from eight trunk and lower-limb muscles, normalized to maximum voluntary contraction. Jumps were segmented into pre-squat and extension phases, and YBT into squat and recovery phases. Statistical analyses employed normality and homogeneity tests, with independent-samples t-tests, Welch’s tests, or Mann–Whitney U tests as appropriate (α = 0.05).

**Results:**

No significant group differences emerged in overall FMS or YBT reach distances; however, martial artists scored higher on the Trunk Stability Push-up (p = 0.013). Sprinters achieved greater CMJ height and relative peak power (p < 0.05, d ≈ 0.83–0.87), whereas SJ outcomes did not differ. sEMG analysis showed higher rectus femoris and lateral gastrocnemius RMS and greater rectus abdominis iEMG (pre-squat) in martial artists, while sprinters exhibited higher erector spinae and gluteus maximus iEMG. During YBT, sprinters relied on rapid spinal extensor activation with partial gluteus medius compensation, whereas martial artists demonstrated integrated core–lower-limb coordination.

**Conclusion:**

Martial artists and sprinters exhibit comparable functional movement and dynamic balance but diverge in core stability and neuromuscular strategies. Martial arts training enhances multiplanar core stability and coordinated muscle recruitment, while sprinting emphasizes stretch–shortening-cycle based explosive output and trunk extensor reliance. These findings provide evidence for targeted conditioning and potential crossdisciplinary training applications.

## 1 Introduction

Postural control is widely recognized as a prerequisite for all forms of human movement. It relies on the central nervous system’s ability to continuously integrate visual, vestibular, and proprioceptive information to maintain the body’s center of mass (COM) over the base of support (Ivanenko and Gurfinkel, 2018). This process relies heavily on the coordinated activity of the neuromuscular system, which ensures efficient sensory processing, accurate motor command execution, and dynamic stability through multi-muscle coordination (De La Motte et al., 2015; Gorgy et al., 2008; Ivanenko and Gurfinkel, 2018). Proficient postural regulation is critical not only for maintaining balance under static conditions but also for facilitating smooth transitions into dynamic movements such as running, jumping, and throwing (Paillard and Noé, 2015).

In competitive sports, postural control is a key determinant of athletic performance and injury prevention (Paillard, 2019; Zemková and Zapletalová, 2022). For sprinters, maintaining COM alignment during the start and acceleration phases is essential to optimize force transmission and running economy (Kubo et al., 2011; Sado et al., 2016; Weyand et al., 2000). Research further shows that the erector spinae, rectus abdominis, and external oblique muscles are rapidly recruited in response to external perturbations, enabling sprinters to maintain trunk alignment and movement efficiency under sudden loading (Glofcheskie and Brown, 2017). Similarly, gymnasts are often considered the benchmark for advanced postural control due to their ability to stabilize under extreme mechanical demands (Hrysomallis, 2011). However, recent studies have shown that Chinese martial arts practitioners demonstrate enhanced postural stability, as reflected by smaller Center of pressure (COP) and COM excursions and faster recovery responses following postural perturbations (Bu et al., 2010; Mustafa et al., 2022).

Chinese martial arts training is distinctive in its multi-planar, multi-directional sequences of movement, which combine jumps, rotations, landings, and complex transitions. These sequences demand simultaneous control of muscular strength, explosive power, core stability, proprioception, and neuromuscular coordination (Guo and Choosakul, 2024; Yan et al., 2022). Beyond physical execution, traditional principles such as “relax and sink the center of gravity” and “seek stillness in motion” emphasize integrated sensorimotor regulation and heightened proprioceptive awareness. Such practices share conceptual parallels with modern functional training, which prioritizes proprioception, joint stability, and dynamic balance through sport-specific tasks. Yet, despite this potential overlap, the role of martial arts as a conditioning modality for athletic postural control has not been systematically evaluated. Most existing research has instead focused on the health benefits of martial arts in older adults or clinical populations (Lee et al., 2015; Wang et al., 2025), leaving a critical gap in understanding their mechanisms and applications within high-performance sport.

Another gap concerns the methodological limitations of prior comparative studies. Many investigations of postural control have relied on static balance tests or global COP metrics, which may not adequately capture the complexity of neuromuscular strategies in elite athletes. Recent advances using surface electromyography (sEMG) allow for task-specific analysis of muscle activation patterns, offering deeper insights into how athletes from different training backgrounds regulate stability under dynamic conditions (Paillard, 2019). Addressing these gaps is crucial. Martial arts and sprinting represent two distinct paradigms of athletic training: the former emphasizes controlled multi-directional movements and integrated whole-body coordination, while the latter develops explosive stretch–shortening-cycle performance and trunk extensor reliance. Both place substantial demands on postural regulation but likely foster divergent neuromuscular strategies. Comparative evidence can therefore not only clarify the adaptive mechanisms shaped by long-term training but also inform conditioning practices and potential cross-disciplinary transfer of training methods.

Accordingly, this study aims to compare the postural control characteristics of elite martial arts athletes and sprinters by integrating functional performance measures with sEMG analysis. Specifically, we examine differences in core stability, dynamic balance, explosive power, and neuromuscular activation strategies. We hypothesize that martial artists, despite training under traditional paradigms, will demonstrate comparable levels of dynamic balance and core stability to sprinters, but will rely on distinct neuromuscular control strategies shaped by their sport-specific demands. Findings are expected to provide a scientific basis for optimizing postural control training and highlight the potential value of martial arts as a functional conditioning tool in modern athletic preparation.

## 2 Methods

### 2.1 Participants

Forty national level 1 athletes were initially recruited from Beijing Sport University (19 martial arts, 21 sprinters). To control intra- and inter-group variability, individuals with marked discrepancies in training years, competitive level, age, height, or body mass were excluded, leaving 16 athletes per group. To reduce heterogeneity, only martial arts athletes specializing in fist forms (Tai Chi, Changquan, Nanquan) were included. Training years (Martial Arts: 7.69 ± 1.92; Sprint: 6.88 ± 1.45), competitive level, and age (Martial Arts: 22.19 ± 1.22; Sprint: 21.75 ± 1.65) did not differ between groups. Table 1, owing to the distinct demands of martial arts and sprinting, group differences in height (Martial Arts: 175.13 ± 7.06 cm; Sprint: 181.81 ± 5.24 cm) and BMI (Martial Arts: 24.08 ± 1.81; Sprint: 21.72 ± 1.30) were unavoidable; these variables were included as covariates in subsequent analyses when appropriate. Training volumes were comparable between groups, but emphases differed: martial arts athletes typically completed 4–5 technical sessions per week (3 h each) and 2 conditioning sessions (1.5 h each), mainly for general fitness maintenance, whereas sprinters completed 4–5 technical sessions (1.5 h each) and 2–3 conditioning sessions (2 h each), emphasizing strength and explosive power development. All participants were in systematic training (>3 sessions per week), reported no major musculoskeletal injuries, and had not undergone surgery within the previous 6 months. They were instructed to refrain from strenuous exercise, smoking, alcohol, and caffeine for at least 48 h prior to testing. Those reporting insufficient sleep or non-compliance with pre-test instructions were excluded. Written informed consent was obtained after participants were fully informed about the study objectives, procedures, risks, and benefits. The study was approved by the Institutional Review Board of the Chinese Institute of Sports Science and adhered to the Declaration of Helsinki.

**TABLE 1:** Description of the participants’ characteristics (Mean ± SD).

Parameter	Martial arts (*N*= 16)	CV%	Sprint (N = 16)	CV%	*p*
Activity level	Level 1	-	Level 1	-	-
Training years	7.69 ± 1.92	24.95	6.88 ± 1.45	21.08	0.287
Age (yrs)	22.19 ± 1.22	5.50	21.75 ± 1.65	7.59	0.423
Height (cm)	175.13 ± 7.06	4.03	181.81 ± 5.24	2.88	0.005
BMI (kg·m^2^)	24.08 ± 1.81	7.52	21.72 ± 1.30	5.99	<0.001

Level 1: national level 1.

### 2.2 Design and procedures

All tests were conducted in a laboratory setting. Upon arrival, participants performed a standardized warm-up, followed by the test protocol administered in a Latin square order to minimize order effects. A 5-minute rest interval was provided between tasks.

**FIGURE 1:**
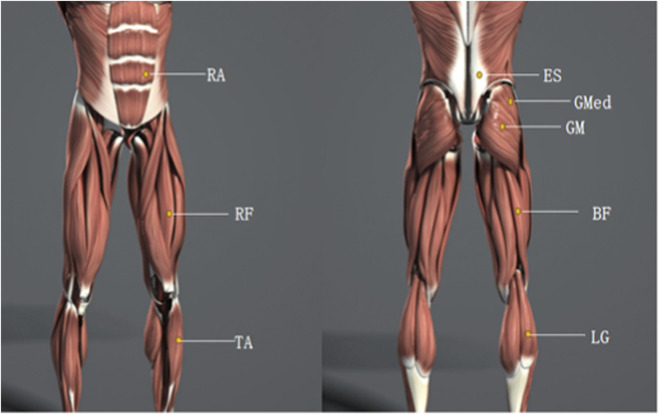
Electrode placement and anatomical localization of surface EMG recording sites. ES, Erector Spinae; RA, Rectus Abdominis; GM, Gluteus Maximus; GMED, Gluteus Medius; RF, Rectus Femoris; BF, Biceps Femoris; TA, Tibialis Anterior; LG, Lateral Gastrocnemius.

Following the CEDE-Check protocol (Besomi et al., 2024), sEMG data were collected during the countermovement jump (CMJ), squat jump (SJ), and Y-Balance Test (YBT) using the Delsys Trigno wireless system (Delsys Inc., Natick, MA, USA) at a sampling rate of 2000 Hz. Skin preparation involved shaving and cleaning the target sites with alcohol swabs. Electrodes were placed according to SENIAM guidelines over the rectus abdominis (RA), erector spinae (ES), gluteus maximus (GM), gluteus medius (GMed), rectus femoris (RF), biceps femoris (BF), tibialis anterior (TA), and lateral gastrocnemius (LG), and secured with medical tape (Figure 1).

The specific test requirements are as follows:

#### 2.2.1 Functional movement screen (FMS)

Seven tasks were administered following standardized FMS protocols. Each task was scored from 0 to 3, yielding a maximum of 21 points. Assessments were conducted by certified FMS evaluators to ensure reliability.

#### 2.2.2 Vertical jump tests

Participants performed three maximal CMJs and three SJs with 2-min rest intervals. Trials were valid only if full leg extension was achieved during flight, landings occurred fully within the force plate (Kistler 9281CA, Switzerland, 1,000 Hz), and no arm or trunk compensations were observed. The mean of three valid attempts was retained for analysis.

#### 2.2.3 Dynamic balance (Y-balance test)

Participants stood on the dominant leg and extended the contralateral leg in the anterior, posteromedial, and posterolateral directions, returning to the start position after each reach. Trials were invalid if the stance foot moved or the reaching foot touched the ground. Three successful attempts per direction were collected, with 30-s rest between trials. The mean reach distance was used for analysis.

### 2.3 Data processing

Raw sEMG signals were band-pass filtered (20–450 Hz, second-order Butterworth), rectified, and smoothed using a low-pass filter (5 Hz, fourth-order Butterworth) in MATLAB 2024a. Root mean square (RMS) values were calculated to estimate muscle activation amplitude. Normalization was performed using the mean RMS of the middle 3 s of maximum voluntary contraction (MVC) trials, and task-related RMS was expressed relative to MVC. To synchronize the EMG with the movement data, we placed an auxiliary EMG sensor within the camera’s field of view; data acquisition began when the sensor’s LED stopped flashing. Jumps were divided into pre-squat and extension phases, while YBT was segmented into squat and recovery phases, using the pelvis’s lowest point as the phase boundary.

### 2.4 Statistical analysis

All statistical analyses were performed using SPSS version 27.0. Data are presented as mean ± standard deviation (Mean ± SD). The Shapiro–Wilk test was used to assess normality, and Levene’s test evaluated homogeneity of variances. For variables that met both normality and homogeneity assumptions, between-group comparisons were conducted using independent-samples Student’s t-tests. If a variable was normally distributed but exhibited unequal variances, Welch’s corrected t-test was applied. Non-normally distributed data were compared with the Mann–Whitney U test. Effect sizes were calculated for all tests: Cohen’s d was reported for t-tests (with |d| = 0.2 indicating a small effect, |d| = 0.5 a medium effect, and |d| = 0.8 a large effect), while the r statistic was used for the Mann–Whitney U test (interpreted as |r| = 0.1 small, |r| = 0.3 medium, and |r| = 0.5 large). Statistical significance was set at α = 0.05.

## 3 Results

The outcomes of the Functional Movement Screen (FMS) are summarized in Table 2. No significant group differences were observed in overall performance on the foundational movement tasks (p > 0.05). However, martial arts athletes achieved significantly higher scores than sprinters in the Trunk Stability Push-up (p < 0.05).

**TABLE 2:** Total score of FMS test and detailed scores of each test (Mean ± SD).

FMS Items	Martial arts	Sprint	*p*
FMS total score	17.81 ± 1.80	16.88 ± 2.22	0.199
Deep squat	2.69 ± 0.48	2.44 ± 0.73	0.465
Hurdle step	2.25 ± 1.06	2.44 ± 0.63	0.723
In-line lunge	2.56 ± 0.51	2.47 ± 0.52	>0.999
Shoulder mobility	2.75 ± 0.58	2.56 ± 0.73	0.683
Active straight-leg raise	3.00 ± 0.00	2.75 ± 0.45	0.109
Trunk stability push-up	2.56 ± 0.89	2.25 ± 0.58	0.013*
Rotary stability	2.00 ± 0.00	2.00 ± 0.00	>0.999

*p < 0.05; **p < 0.001.

Table 3 presents the results of the vertical jump and dynamic balance assessments. In the CMJ, sprinters demonstrated significantly greater relative peak power and jump height than martial arts athletes (p < 0.05, |d| = 0.827–0.874, large effects). In contrast, SJ outcomes did not differ between groups (p > 0.05), and no significant differences were found in YBT reach distances.

**TABLE 3:** Vertical jump performance and Y-balance test performance (Mean ± SD).

Variables	Martial arts	Sprint	*p*	ES	
				*d*	95% CI
CMJ					
Relative peak power (W/kg)	50.20 ± 2.55	53.73 ± 5.47	0.026*	0.827	[−1.54, −0.10]
Jump height (cm)	39.67 ± 2.87	43.69 ± 5.83	0.022*	0.874	[−1.60, −0.14]
SJ					
Relative peak power (W/kg)	48.37 ± 4.04	49.33 ± 3.81	0.494	0.245	[−0.94, 0.45]
Jump height (cm)	36.73 ± 5.34	37.37 ± 6.14	0.747	0.115	[−0.81, 0.58]
Y-balance					
Anterior	73.45 ± 5.91	71.42 ± 5.44	0.32	0.357	[−0.34, 1.05]
Posteromedial	114.95 ± 7.52	112.17 ± 8.13	0.701	0.137	[−0.56, 0.83]
Posterolateral	116.03 ± 9.11	117.46 ± 12.45	0.713	0.131	[−0.82, 0.56]

*p < 0.05; **p < 0.001.

**FIGURE 2:**
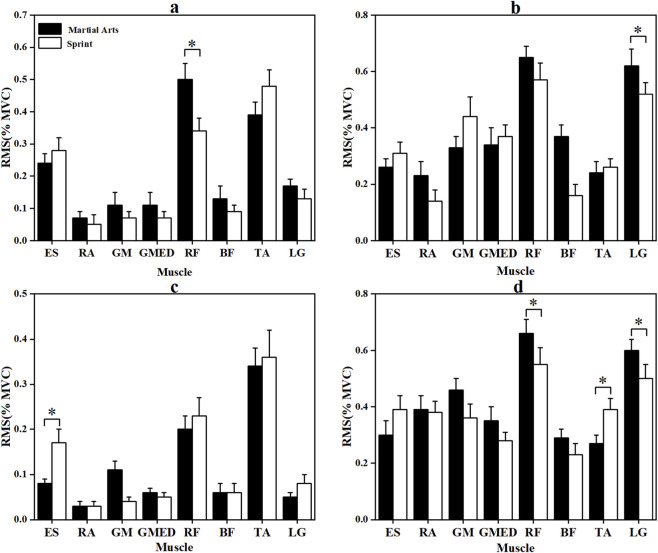
RMS (%MVC) of the two groups of Participants in the pre-squat and extension phases of CMJ and SJ jumps. *: p < 0.05, **: p < 0.001; error bars are standard errors. **(a,b)** are the pre-squat stage and the extension stage of CMJ, respectively; **(c,d)** are the pre-squat stage and the extension stage of SJ, respectively. Muscle abbreviations are shown in Figure 1.

**FIGURE 3:**
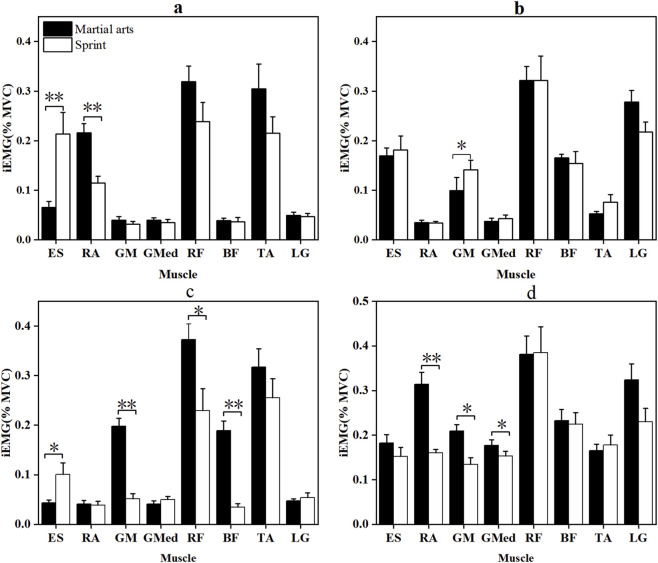
iEMG (%MVC) of the two groups of Participants in the pre-squat and extension phases of CMJ and SJ jumps. *: p < 0.05, **: p < 0.001; error bars are standard errors. **(a,b)** are the pre-squat stage and the extension stage of CMJ, respectively; **(c,d)** are the pre-squat stage and the extension stage of SJ, respectively. Muscle abbreviations are shown in Figure 1.

Figures 2, 3 depict the RMS and integrated EMG (iEMG) values during the CMJ and SJ phases. In the CMJ, martial arts athletes exhibited significantly higher RMS values for the RF (p = 0.016, |d| = 0.883) and LG (p = 0.030, |d| = 0.785), as well as greater RA iEMG in the pre-squat phase (p < 0.001, |r| = 2.889). Conversely, sprinters showed higher ES (p < 0.001, |r| = 0.774) and GM (p = 0.025, |r| = 0.410) activation. During the SJ, sprinters presented significantly higher RMS values for the TA (p = 0.011, |r| = 0.444) and ES iEMG during extension (p < 0.05, |r| = 0.487). Martial arts athletes, however, displayed significantly greater RF (p = 0.001, |d| = 1.244) and LG (p = 0.025, |r| = 0.403) RMS activation, along with higher pre-squat GM (p < 0.001, |d| = 2.627), RF (p = 0.006, |r| = 0.493), and BF activity (p < 0.001, |r| = 0.850). Additionally, RA RMS in the extension phase was significantly greater in martial arts athletes (p < 0.001, |d| = 1.850). In contrast, sprinters showed significantly higher GM, GMed, and other muscle activations across both phases, with medium-to-large effect sizes.

**FIGURE 4:**
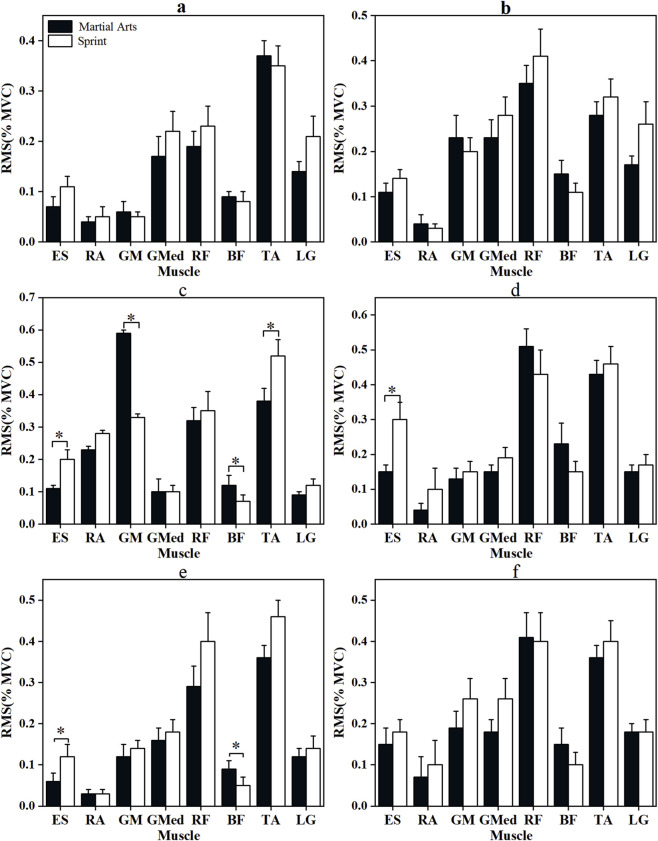
RMS (%MVC) of the two groups of Participants during squatting and recovery phases in all directions of Y Balance Test. *: p < 0.05, **: p < 0.001; error bars are standard errors. **(a,b)** are the squatting stage and recovery stage of the anterior direction of YBT, respectively; **(c,d)** are the squatting stage and recovery stage of the posteromedial direction of YBT, respectively; **(e,f)** are the squatting stage and recovery stage of the posterolateral direction of YBT, respectively. Muscle abbreviations are shown in Figure 1.

**FIGURE 5:**
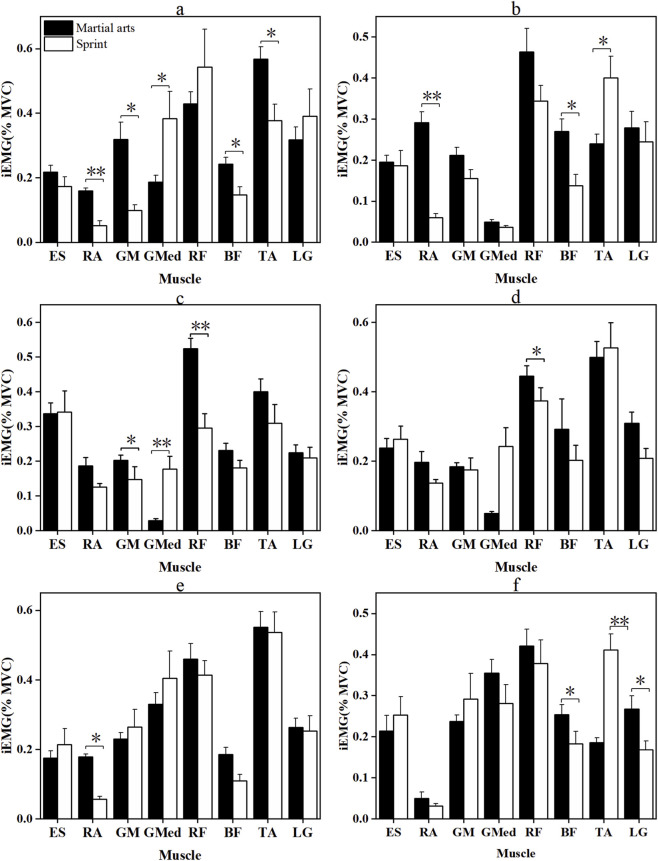
iEMG (%MVC) of the two groups of Participants during squatting and recovery phases in all directions of Y Balance Test. *: p < 0.05, **: p < 0.001; error bars are standard errors. **(a,b)** are the squatting stage and recovery stage of the anterior direction of YBT, respectively; **(c,d)** are the squatting stage and recovery stage of the posteromedial direction of YBT, respectively; **(e,f)** are the squatting stage and recovery stage of the posterolateral direction of YBT, respectively. Muscle abbreviations are shown in Figure 1.

Figures 4, 5 illustrate the RMS and iEMG activities during the squat and recovery phases of the YBT in the anterior, posteromedial, and posterolateral directions.

In the anterior direction, Martial arts athletes showed significantly greater activation of RA, GM, BF, and TA during the squat phase (p < 0.05, |d| = 1.036–1.316), and higher RA and BF activity during recovery (p < 0.05, |d| = 1.155–2.775). In contrast, sprinters exhibited significantly greater GMed activation during squatting (p < 0.05, |d| = 0.853) and higher TA activation during recovery (p < 0.05, |d| = 1.034).

In the posteromedial direction, printers demonstrated greater ES and TA RMS values during squatting (p < 0.05, |d| = 1.148; |r| = 0.423), while martial arts athletes showed higher BF activation (p < 0.05, |r| = 0.680). iEMG analysis revealed greater LG activation in martial arts athletes (p < 0.05, |d| = 0.823), whereas GMed activity was significantly higher in sprinters (p < 0.05, |r| = 0.531). During recovery, sprinters demonstrated significantly greater ES RMS (p < 0.05, |r| = 0.501) and GMed iEMG (p < 0.001, |d| = 1.532), while martial arts athletes displayed higher RA and RF activation (p < 0.05, |r| = 0.478; |d| = 1.633).

In the posterolateral direction, Sprinters exhibited higher ES RMS during squatting (p < 0.05, |r| = 0.351), whereas martial arts athletes displayed significantly greater BF RMS (p < 0.05, |r| = 0.412) and RA and BF iEMG (p < 0.05, |d| = 0.976–3.614). During recovery, martial arts athletes showed greater BF and LG activation (p < 0.05, |r| = 0.380; |d| = 0.886), while sprinters demonstrated higher TA activity (p < 0.001, |d| = 1.154).

## 4 Discussion

This study systematically compared elite Chinese martial arts athletes and sprinters regarding functional movement patterns, lower-limb explosive power, dynamic balance ability, and task-specific muscle activation. The aim was to explore how varying training backgrounds influence postural control. The results indicated significant differences between the two groups in core stability, power output, and neuromuscular control strategies, thereby confirming the specific shaping effect of specialized training on the development of postural control.

In the FMS, no significant differences between groups were detected for any items except for the Trunk Stability Push-up (p > 0.05), suggesting comparable postural control in fundamental movement patterns for both groups (Moran et al., 2017). This result was consistent with expectations because both the complex multi-planar transitions of Chinese martial arts and the precise center-of-mass control required for sprint starts and acceleration depend on stable, high-quality fundamental movement patterns, a key capability shared among elite athletes (Hrysomallis, 2011; Paillard, 2014; 2017).

A key finding of this study was the pronounced difference between the two groups in postural control dimensions linked to core stability. This advantage may result from the sustained emphasis of martial arts training on coordinated control of the core, pelvis, and lower-limb kinetic chain. Martial arts training entails extensive static and dynamic postural control, gradual center-of-mass transitions, precise weight redistribution, and complex kicking and jumping techniques. These movement patterns have been demonstrated to enhance spinal stability, hip flexor flexibility, and pelvic support capacity (Wehner et al., 2022; Zhang et al., 2023; Zou et al., 2017). Consequently, this training system promotes the development of core stability and elicits specific neuromuscular adaptations. From a neuromuscular control perspective, gradual shifts in the center of mass, combined with the breathing–core synergy mechanism, contribute to increased intra-abdominal pressure (IAP) and enhanced anticipatory postural adjustments (APAs). Additionally, the holistic philosophy of “integration of form and mind” reinforces coordinated core–pelvis–limb function, thereby enabling efficient force transmission and improved postural stability.

The sEMG results further confirmed a core-dominant postural control strategy among martial arts athletes. During the eccentric phase of the CMJ and the concentric phase of the SJ, martial arts athletes showed significantly higher iEMG activity in the RA than did sprinters. This finding indicates a preference for stability enhancement via feedforward core activation and elevated IAP during explosive tasks. For instance, in isometric resistance tasks, IAP is elevated through a breathing–core coupling mechanism (Walters, 2020), thereby enhancing trunk stiffness and proximal stability. This approach reduces proximal energy loss and improves force transmission efficiency from the core to the lower limbs (Kocjan et al., 2018; Seo et al., 2024; Yu et al., 2022), consistent with martial arts principles emphasizing integration of form and mind and breath-guided movement (Li et al., 2018).

Conversely, sprinters displayed significantly higher jump height and relative peak power in the CMJ, accompanied by greater iEMG activity in the ES and GM. These findings indicate reliance on a hip-extension–dominant posterior-chain activation strategy. This pattern is consistent with adaptations induced by high-load resistance training and the stretch–shortening cycle (SSC), including an increased proportion and cross-sectional area of fast-twitch fibers, enhanced neural conduction efficiency, and greater synchronization of high-threshold motor unit recruitment (Aagaard et al., 2002; Cormie et al., 2011; Donahue et al., 2021). The hip-extension–dominant recruitment pattern closely aligns with the demands of sprint starts and acceleration, which rely heavily on posterior-chain muscles such as the GM and ES for rapid extension and anti-flexion control. This pattern reflects sport-specific neural drive adaptations. Correlation analysis revealed moderate associations between RMS amplitudes of the LG and RA during the countermovement phase of the CMJ and both jump height and relative peak power. These results suggest that the LG and RA act as efficiency links within SSC movements. Pre-activation of the LG regulates Achilles–muscle–tendon complex stiffness, enhancing elastic energy return and power output, whereas feedforward activation of the RA maintains proximal stiffness and supports efficient longitudinal force transmission. By comparison, martial arts training involves fewer high-intensity SSC stimuli and places greater emphasis on movement control and flexibility. Consequently, although martial arts athletes exhibit higher RMS amplitudes during explosive tasks, this activation may not translate into proportional force output at maximal shortening velocity, possibly explaining their lower CMJ height and power relative to sprinters.

However, no significant differences in jump height or relative peak power were detected between the two groups in the SJ test. This finding indicates that when the contribution of elastic energy from the SSC is excluded, both groups exhibit comparable maximal concentric force-generating capacity. Therefore, the performance advantage of sprinters lies in their efficient utilization of the SSC rather than in superior absolute concentric strength. Thus, their superior CMJ performance can be attributed to effective integration of eccentric–concentric transitions and exploitation of tendon elastic recoil. Notably, during the concentric phase of the SJ, martial arts athletes showed higher RMS values in the RA and LG, together with greater iEMG activity in the RA, GM, RF, and BF. This activation pattern suggests a compensatory strategy for maintaining postural control and facilitating force transmission through multi-muscle coactivation and core stabilization when elastic energy support is absent. This strategy is consistent with the long-established “stability-first” principle in martial arts training, emphasizing coordination and control to improve overall movement efficiency rather than maximizing individual-muscle power output (Fong et al., 2017). Hence, the superior CMJ performance of sprinters can be attributed to efficient SSC utilization; however, this advantage diminishes in the SJ, where SSC contribution is absent, leading to comparable performance between groups (Donahue et al., 2021). Martial arts athletes attain an optimal balance of functional stiffness through feedforward core activation and multi-muscle coordination, thereby ensuring efficient force transmission while maintaining movement flexibility and stability (Kubo et al., 2021; Kubo et al., 2017).

In dynamic balance tasks such as the YBT, group differences in control strategies became more pronounced. During both the squatting and return phases, martial arts athletes showed greater activation of core and posterior-chain muscles across the anterior, posteromedial, and posterolateral directions. They especially activated RA, BF, and LG. This pattern indicates a complementary feedforward–feedback control mechanism. In the descent phase, early RA activation increases IAP and trunk stiffness, thereby providing stable proximal support for lower-limb motion (Hodges and Gandevia, 2000; Walters, 2020). The simultaneous co-contraction of the BF and LG generates moderate joint stiffness toward the end of the movement, facilitating postural readiness (Bhanot et al., 2019; Marcolin et al., 2022). This feedforward activation pattern resembles APAs and provides an active stabilizing foundation for movement initiation (Hodges and Richardson, 1997). During recovery, sustained RA and BF activation maintains pelvic and spinal stability, resists external perturbations, and enables rapid center-of-mass realignment. The LG modulates terminal ankle stiffness through plantarflexion–inversion coupling, allowing precise “ankle-strategy” control (Marcolin et al., 2022).

In contrast, sprinters relied predominantly on localized activation of the ES and GMed. Specifically, in the posteromedial recovery phase, sprinters exhibited higher iEMG activity in the GMed (p < 0.05), indicating frontal-plane hip-abduction compensation to stabilize center-of-mass realignment. This strategy corresponds to the sagittal-plane–dominant training pattern of sprinters, which emphasizes high-speed extension and stiffness regulation (Bramah et al., 2024; Morin et al., 2015; Yoshimoto et al., 2025). Continuous ES activation reflects a requirement to maintain spinal anti-flexion stiffness and upright trunk posture throughout forward-reach and recovery phases. This postural-control pattern arises from posterior-chain adaptations cultivated through sprinting to counter high-speed extension and ground-reaction forces. However, this strategy may not reflect superior dynamic stability but rather a protective compensatory response to non-sagittal-plane perturbations. Moreover, balance-training effects and performance transfer are task-specific: the closer the mechanical and control characteristics between training and testing, the greater the performance enhancement and neuromuscular adaptation (Rizzato et al., 2025; Rizzato et al., 2023). In other words, the long-term emphasis in martial arts training on multi-directional weight shifting, rhythm variation, and multi-joint coordinated output enables athletes to exhibit stronger core–lower-limb coactivation during multi-planar dynamic tasks.

In summary, although Chinese martial arts athletes exhibited lower absolute power output in the CMJ than sprinters, their greater core stability, multi-muscle coordination, and multi-planar dynamic balance underscore the distinctive value of traditional Chinese martial arts for enhancing proximal stability and holistic coordination. Several limitations must be acknowledged. (1) The cross-sectional design limits causal inference and prevents full separation of self-selection and training effects. (2) The small, male-dominated sample restricts generalizability; although subdiscipline and anthropometric variables (e.g., height, BMI) were controlled, residual confounding may persist. (3) Mechanical properties of the muscle–tendon unit (e.g., assessed by ultrasound), IAP, trunk stiffness, and three-dimensional kinetic coupling were not directly measured. Future investigations should further refine and classify martial-arts-specific training protocols to compare subdiscipline- and dose-dependent effects. Randomized-controlled and longitudinal designs integrating tendon/fascicle ultrasound, IAP monitoring, kinetic assessment, and EMG-synergy modeling (including structural and temporal parameters) are recommended to verify—in both youth and elite populations—the dual impact of incorporating martial-arts elements into strength- and speed-training programs on performance enhancement and injury-risk reduction.

## 5 Conclusion

This study compared elite Chinese martial arts athletes and sprinters to elucidate how long-term training shapes neuromuscular control strategies underlying core stability, explosive power, and dynamic balance. Martial artists exhibited superior trunk stability and multi-muscle coactivation during balance and control tasks, whereas sprinters relied more on posterior-chain musculature for power generation during stretch–shortening movements. These distinct yet complementary adaptations illustrate how stability-oriented and force-oriented training develop different aspects of neuromuscular efficiency.

From a practical standpoint, martial arts–inspired drills emphasizing controlled center-of-mass transitions, coordinated breathing, and core–limb coactivation can be incorporated into athletic conditioning to complement traditional power training. Such integrative approaches may enhance explosive performance, improve balance recovery under perturbations, and reduce energy loss during force transmission—benefiting sports such as sprinting, gymnastics, and team-based disciplines. In summary, martial arts training offers a functional model for developing stability–mobility synergy, providing actionable insights for optimizing strength and conditioning programs.

## Data Availability

The raw data supporting the conclusions of this article will be made available by the authors, without undue reservation.
